# One-Pot Synthesis of GeAs Ultrafine Particles from Coal Fly Ash by Vacuum Dynamic Flash Reduction and Inert Gas Condensation

**DOI:** 10.1038/s41598-017-03398-1

**Published:** 2017-06-16

**Authors:** Lingen Zhang, Zhenming Xu

**Affiliations:** School of Environmental Science and Engineering, Shanghai Jiao Tong University, 800 Dongchuan Road, Shanghai, 200240 People’s Republic of China

## Abstract

Ge-monopnictides (GeAs) plays critical role in high-tech industry, especially in the field of advanced optical devices and infrared. As a secondary material, coal fly ash could be further recycled to retrieve germanium and prepare GeAs material with high added values. Hence, the aim of this paper is to propose a one-pot synthesis that uses vacuum flash reduction and inert-gas consolidation method to prepare GeAs ultrafine particles. Germanium in coal fly ash can be successfully recycled; simultaneously, GeAs ultrafine particles were prepared. Separation principle and feasibility of this process was discussed. Temperature, carrier gas flow rate and system pressure were the major factors on formation, morphology and distribution of particle size of GeAs ultrafine particles. A three steps synthetic mechanism was clarified, namely, thermal rupture of coal fly ash and release of GeO_2_ and As_2_O_3_, the gas-solid phase reaction of GeO_2_, As_2_O_3_ and coke to generate metallic Ge and As in vacuum flash reduction. Meantime, GeAs were produced in the gas phase reaction. Finally, GeAs ultrafine particles were obtained by carrier gas condensation. In short, this research developed a practical and environment-friendly one-pot synthesis to recycle germanium in coal fly ash and prepare GeAs ultrafine particles with high added values.

## Introduction

Over the past decade, germanium and Ge-monopnictides such as GeP, GeAs and GeAs_2_ has been applied in the manufacture of advanced electronic, optical devices, and infrared due to its ultrahigh-efficiency and special performance^1^. However, with growing demand for germanium in the field of high technology, the resources of germanium are extremely scarce in worldwide. Ge does not form specific ore deposits in the nature and usually occurs as a substitute for Zn in sphalerite or in Ag, Fe, Cu sulphides ores such as argyrodite, renierite^2, 3^. It is also found as an oxide, mainly argutite (tetragonal-GeO_2_), usually substituting for SiO_2_ in silicates^3, 4^. In worldwide, the proven reserves of germanium are 8600 tons, which is only 1/10 of the reserves of gold. The global reserves of germanium would be exhausted after about 40 years with exploiting rate of 200 t/year^5^. Germanium has been categorized in the one of 14 extreme shortages of mineral resources by the European Commission^6^. China has been leading the global production of germanium and supplied more than 60% of germanium raw material worldwide resulting from weak deep-processing to produce high value-added germanium products^7^. Hence, recycling and production of high value-added germanium products from wastes have both important significance and urgent demand.

Coal fly ash generated in coal combustion process is considered as industrial waste. Huge environmental and economic costs need to be paid to dispose them. In recent years, there are some new approaches for recycling of coal fly ash with harmless treatment and high value-added utilization, such as synthesis of zeolites/geopolymers, fire resistant materials, ceramic manufacture, recovery of valuable metals, and agricultural applications^8–10^. Recovery of valuable metals is a feasible approach for comprehensive utilization of coal fly ash. In Yunnan and Inner Mongolia of China, a certain type of lignite contains rich germanium resource. Germanium in coal fly ash can be concentrated to 0.1 wt.–wt. % when the coal is burned under proper conditions. The content of germanium in coal fly ash is ten or dozens of times higher than feed coal. Hence, this part of coal fly ash can be totally taken advantage of as resources. Utilizing germanium in coal fly ash with high added values is a significant work. It can be take full advantage of coal fly ash, but also avoid waste of scarce resources.

Current techniques have been reported to separate germanium including precipitation with tannin^11, 12^, distillation of strong acid, ion exchange/flotation^13, 14^, adsorption onto activated carbon^15^ and solvent extraction^16, 17^. Xu *et al*.^18^ adopted tannin precipitation to recycle germanium from slag with 5% oxalic acid and 10% aqueous sodium hydroxide adjusting pH 8–9. F. Arroyo Torralvo^13^ recovered germanium from real fly ash leachates by using ion-exchange procedures which based on germanium complexation with catechol (CAT) and followed by the retention of the Ge-CAT complex onto a conventional strongly basic anionic resin (IRA-900). Keisuke Kuroiwa *et al*.^19^ studied recovery of germanium from waste solar panels using ion-exchange membrane and solvent extraction. Although these studies have focused on recycling of germanium resource, environmental improvement are still challenging due to limitations on using large volume of acid/alkali/organic reagent with high concentration. Meanwhile, severe environmental problems might be triggered by solvent leaching since most organic solvents are toxic, flammable or corrosive.

Compared with hydrometallurgy and extraction processes, vacuum metallurgical method has been showed many advantages such as simple technological flow sheet, no or low environmental pollution, and low consumption of raw material and energy^20, 21^. Its principle is that the saturation vapor pressure of metal under the vacuum condition is lower than normal pressure to separate metals, and these metals easy to evaporate into the gas phase^22^. In industry, it has been successfully applied in non-ferrous metal smelting and recycling. Lin *et al*.^23, 24^ applied vacuum dynamic flash reduction to treat arsenic- and antimony-rich anode slime. As_2_O_3_ and Sb_2_O_3_ in anode slime, as soon as produced by reducing the high valence oxides of arsenic and antimony, are evaporated at once and then stop being reduced to metals further. In our previous study, vacuum method has also successfully separated and recycled copper, lead and gallium etc from electronic wastes^25, 26^.

There is a great deal of interest in nanostructured metals due to their unique electrical, chemical and optical characteristics. Ultrafine Ge-monopnictides are one important material that has a great potential to be used as solid-state lighting, sensors, and other new type of electronic and optoelectronic devices^27, 28^. Several methods have been developed for the preparation of nano-sized materials, such as solution-phase chemical reduction^29^, pulsed laser deposition^30, 31^, and chemical or physical vapor deposition^32^. Currently, one way to produce metal nanoparticles is the inert gas condensation (IGC) method. Metal nanoparticles prepared with IGC method have the advantages of narrow particle size distribution and controllable particle size^33, 34^. In light of the present work, it is noteworthy to mention reports of the synthesis of nanoparticle from electrical and electronic equipment (WEEE). Zhang *et al*.^35^ reported a synthesis of lead nanoparticle from waste cathode ray-tube funnel glass using IGC technology and gained purity of lead nanoparticle exceeded 95%. Xiang *et al*.^36^ prepared zinc nanoparticles from spent zinc manganese batteries by vacuum separation technology. In previous works, we have successfully separated germanium from coal fly ash efficiently by vacuum reduction metallurgical process^37, 38^.

Hence, in this study, a novel one-pot synthesis, namely vacuum flash reduction and inert-gas consolidation method was proposed to prepare ultrafine Ge-monopnictides (GeAs) through coal fly ash as germanium source. The aim of this study was to explore the feasibility of this method for preparation of GeAs ultrafine particles. Meantime, the influences for ultrafine Ge-monopnictides preparation, including evaporation percent, morphology and particles size were studied. Finally, a synthetic mechanism of ultrafine Ge-monopnictides is discussed and analyzed to understand the integrated method. In short, this study provides a green, non-polluting and efficient method to recycle germanium from coal fly ash with high value.

## Materials and Methods

### Materials and Chemicals

Coal fly ash enriched germanium from Inner Mongolia province of China was chosen as sample and the reductant used in this study was coke powder (0.3–0.5 mm) in order to adapt to industrial application in the future. The XRF analysis result of germanium-rich coal fly ash was shown in Table 1, which showed the main chemical composition for the coal fly ash sample. The content of germanium in coal fly ash by measurement of chemical method was 9508 mg/kg.

**Table 1:** Main Chemical Composition of Germanium-Rich Coal Fly Ash.

element	C	O	Si	Cl	S	Ca	Fe	Al	As	Zn	Pb	Cr
content (wt. %)	46.3	24.66	5.96	6.24	1.96	6.53	3.57	2.57	0.44	0.28	0.09	0.01

### Principle Analysis

In the process of vacuum flash reduction, there are two main procedures, namely, the first procedure is reduction reaction of GeO_2_ and As_2_O_3_, and the second procedure is vacuum distillation of germanium and arsenic.

Firstly, GeO_2_ and As_2_O_3_ were considered to be reduced to metallic Ge and As under certain temperature and carrier gas flow. Based on thermodynamic data from thermodynamic handbook^39^, the reactions between GeO_2_, As_2_O_3_ and C, and corresponding standard Gibbs free energy changes (Δ_r_G_T_^Θ^) under air condition and exceeding temperature of 1000 K were listed in Eqs 1 and 2:

1 $${{\rm{GeO}}}_{2}({\rm{s}})+{\rm{C}}({\rm{s}})={\rm{Ge}}({\rm{s}})+{{\rm{CO}}}_{2}({\rm{g}}),{{\rm{\Delta }}}_{{\rm{r}}}{{\rm{G}}}^{{\rm{\Theta }}}(1000\,{\rm{K}})=-3.77\,{\rm{KJ}}/{\rm{mol}}$$

2 $${{\rm{As}}}_{2}{{\rm{O}}}_{3}({\rm{s}})+{\rm{C}}({\rm{s}})={\rm{As}}({\rm{s}})+{{\rm{CO}}}_{2}({\rm{g}}),{{\rm{\Delta }}}_{{\rm{r}}}{{\rm{G}}}^{{\rm{\Theta }}}(1000\,{\rm{K}})=-168.43\,{\rm{KJ}}/{\rm{mol}}$$

The reduction reaction between GeO_2_, As_2_O_3_ and C can be taken place when Δ_r_G_T_^Θ^ is negative value. Hence, when temperature exceeded 1000 K, reduction reaction of GeO_2_ and As_2_O_3_ can occur.

Vacuum distillation was based on the different vapor pressure of various metals and their compounds at the same temperature. Metals and their compounds with high vapor pressure and low boiling point can be separated from reaction system, and then be recovered. According to Clausius-Clapeyron equation, the relationship between vapor pressure and temperature can be shown Eq. 3:

3 $$\frac{{\rm{d}}\,\mathrm{ln}\,{{\rm{P}}}_{{\rm{Me}}}}{{\rm{dT}}}=\frac{{{\rm{\Delta }}H}_{({\rm{s}},{\rm{v}})}^{\ast }}{{{\rm{RT}}}^{2}}$$

where P_Me_ is the vapor pressure of substance; ∆H*_(*S, V*)_ is the standard molar enthalpies of evaporation or sublimation; T is temperature; R is the universal gas constant.

Replaced ∆H*_(S,V)_ with ∆_r_H_m_^Θ^, and assumed that ∆_r_H_m_^Θ^ was constant over the temperature. Then, Eq. 3 was integrated to obtain Eq. 4. Constants of A, B and C were determined for each substance.

4 $${\rm{lg}}\,{{\rm{p}}}_{{\rm{Me}}}^{\ast }=-\frac{{{\rm{\Delta }}}_{{\rm{r}}}{{\rm{H}}}_{{\rm{m}}}^{{\rm{\Theta }}}}{2.303{\rm{RT}}}+\frac{{\rm{C}}}{2.303}=\frac{{\rm{A}}}{{\rm{T}}}+{\rm{B}}$$

where p^*^_Me_ is the vapor pressure of substance; ∆_r_H_m_^Θ^ is standard molar enthalpies of evaporation or sublimation. A, B and C are constants determined for each substance.

**Figure 1:**
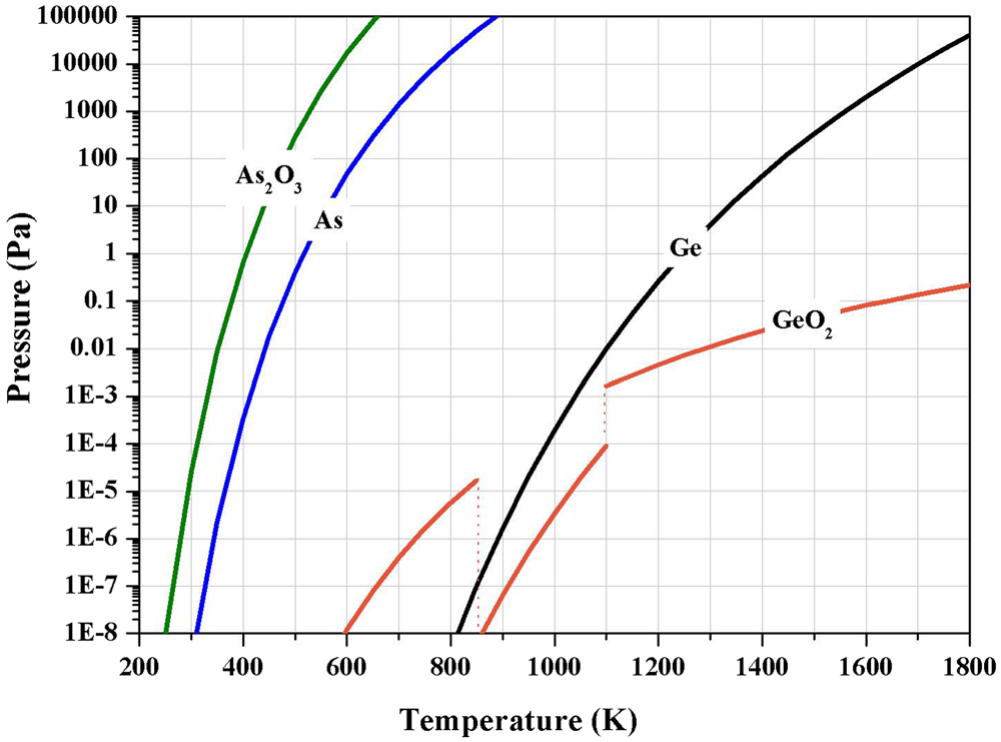
Relationship between saturated vapor pressure of Ge, As and GeO_2_, As_2_O_3_ and temperature.

Figure 1 showed saturated vapor pressure equation of Ge, As, GeO_2_ and As_2_O_3_^40–43^. We can find that As and As_2_O_3_ are easy to evaporate under vacuum condition. The saturated vapor pressure of Ge was higher than that of GeO_2_ after the temperature of 1000 K. This indicated that Ge can be easily evaporated into gas phase and condensed on low temperature zone under vacuum condition. And when Ge and As entered into the gas phase, conjugation reaction under the high temperature can be happen to generate GeAs (as shown in Eq. 5).

5 $${\rm{Ge}}+{\rm{As}}={\rm{GeAs}}({\rm{s}}),{{\rm{\Delta }}}_{{\rm{r}}}{{\rm{G}}}^{{\rm{\Theta }}}(1000\,{\rm{K}})=-83.08\,{\rm{KJ}}/{\rm{mol}}$$

Inert gas consolidation (IGC) method has been widely used in the synthesis of size, shape, and structure-controlled nanoparticles. It also has been considered as the cost-effective and efficient method for large scale cluster synthesis^44, 45^. Synthesis of GeAs ultrafine particles from coal fly ash based on inert-gas consolidation process mainly included precursor gas reaction, surface growth, particle nucleation, coagulation and coalescence^35^. Based on above principle analysis, we adopted a novel one-pot synthesis by vacuum flash reduction and inert-gas consolidation to prepare GeAs ultrafine particles in this study.

### Exploratory Experiment

**Figure 2:**
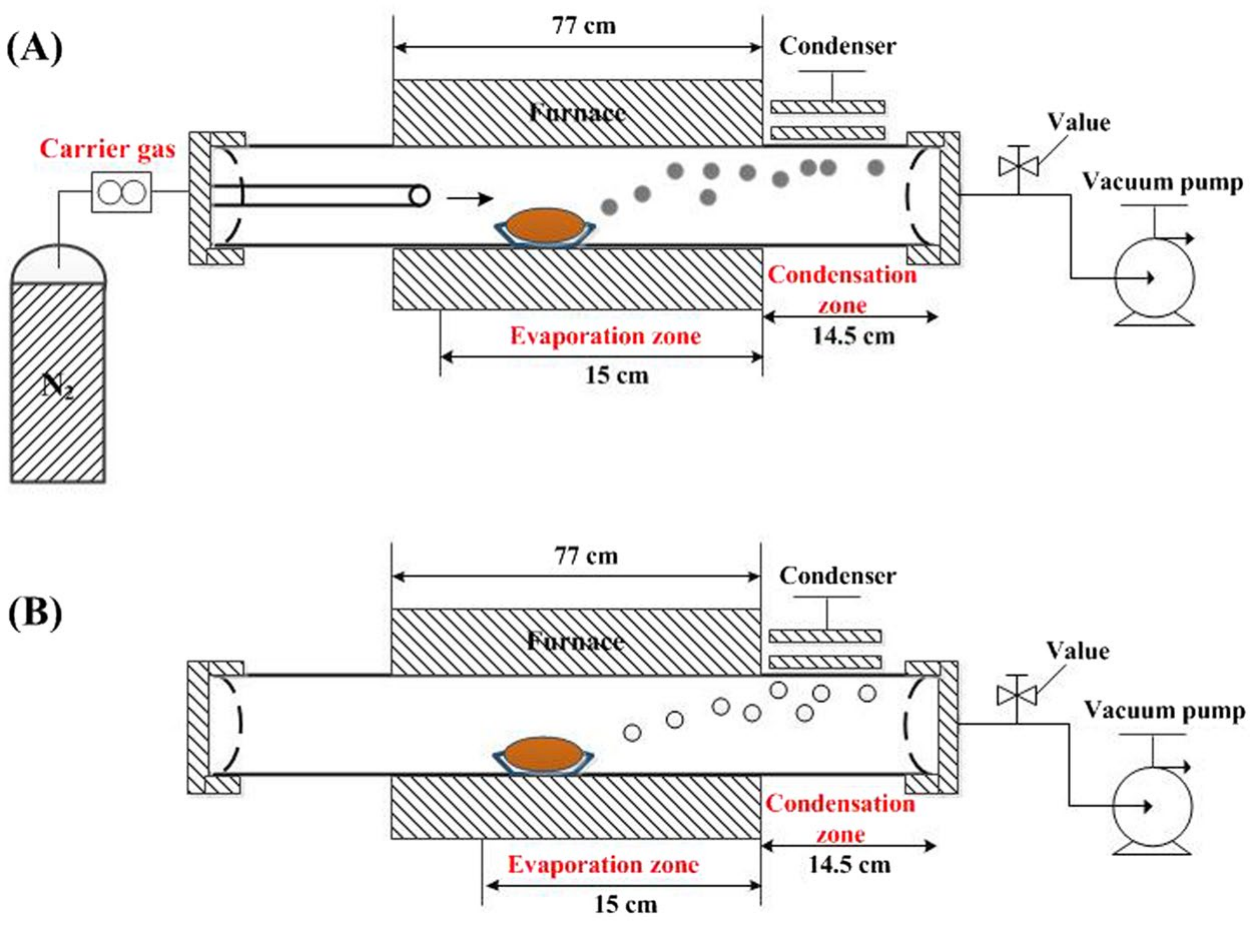
Schematic illustration of (**a**) dynamic state and (**b**) static state of vacuum flash reduction-inert gas condensation.

Vacuum flash reduction and inert gas condensation experiments were carried out in a self-made vacuum tube furnace to recycle and prepare GeAs ultrafine particles from coal fly ash, as shown in Fig. 2. The apparatus consist of four basic units: a temperature controller, inert gas supply system, quartz tube furnace, vacuum pump and condenser. 10 g of coal fly ash mixed with 10 wt. % reductant (coke powders) was loaded in the quartz boat and placed in the center position in the furnace and then the quartz tube reactor was sealed. Opened vacuum pump and kept residue pressure of system was less than 1500 Pa.

**Figure 3:**
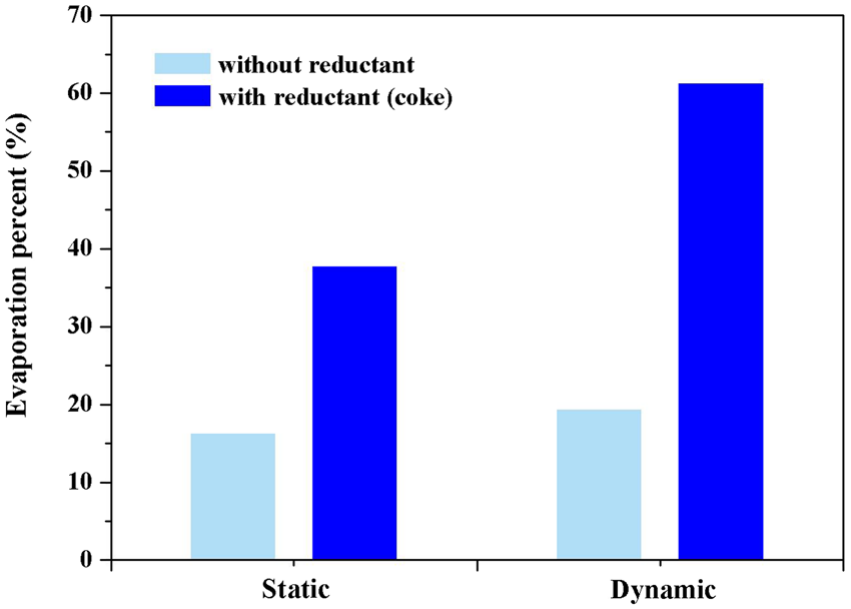
Comparison of dynamic state and static state of vacuum flash reduction.

So called “vacuum dynamic flash reduction” means that inert gas with a certain flow rate was introduced through the pipe inlet into the system with reduction reaction under a certain vacuum degree. In contrary, “static state” means that no flow of inert gas was introduced to the system during the experiment. In the experiments, nitrogen as carrier gas with a certain gas flow rate was passed through the quartz tube furnace. Figure 3 showed the schematic diagram of vacuum flash reduction and inert gas condensation: (A) dynamic state; (B) static state. The residual pressure of system for static state experiments was kept constant at 1300 Pa.

In a period of time, the sample was cooled on the condenser through vacuum flash reduction-inert gas condensation. After the experiments, the residues in the quartz boat was taken, and weighed. The evaporation percent of germanium were calculated through the determination of the content of germanium in residues before and after reaction. The evaporation percent (E) of germanium were calculated by the following formulas (Eq. 6):

6 $${\rm{E}}=\frac{{{\rm{M}}}_{0}\,-\,{\rm{M}}}{\,\,{{\rm{M}}}_{0}}\times 100 \% $$

where, M_0_ and M are the initial and remaining amount of germanium in coal fly ash, respectively.

Two groups of experiments were carried out in static and dynamic state at 1173 K for 40 min with 10 wt. % dosage of reductant or without reductant, respectively. As can be seen from Fig. 3, if without reductant, the evaporation percent of germanium has only 16.3% in static condition, but it reached 37.8% in static condition with 10 wt. % dosages of reductant. Similarly, the evaporation percent of germanium was increased by 41.89% under dynamic state with 10 wt. % reductant dosages, which can reach 61.32%. It can significantly see that vacuum dynamic flash reduction was superior to static state. Hence, vacuum dynamic flash reduction was chose in this study. Meantime, the evaporation percent of germanium from coal fly ash, morphology and distribution of particles size for synthesis of GeAs ultrafine particles were studied under different experimental conditions.

### Analysis Methods

A distillation separation and benzfluorenone spectrophotometric method was introduced for the determination of germanium in raw coal fly ash and residues. For samples of coal fly ash and residues, samples were firstly digested with nitric acid, phosphoric acid, and hydrofluoric acid, and formed digestive solution. And then, digestive solution was distilled with 6 mol/l hydrochloric acid and 3 g KMnO_4_. The distillation temperature was controlled at 363 K and the distillation time was set to 40 min. Finally, colorimetric determination was used with solution of saturated sodium sulfite, dodecyl trimethyl ammonium bromide (0.5%) and benzfluorenone (0.03%).

The collected nanoparticles were characterized by X-ray diffraction (XRD) using the Ni-filtered Cu K radiation on a RigakuD/MAX2500 diffractometer over an angle from 10° to 90°. Then the GeAs ultrafine particles were withstood ultrasonic dispersion treatment in ethanol for 30 min and Transmission Electron Microscopy, Field-emission Scanning Electron Microscopy & Energy Dispersive Spectrometer (TEM Sirion 200 & EDS INCA X-Act, FEI Company, America & Oxford Company, England) was employed to examine the particles size and morphology of GeAs ultrafine particles. Nanometer particle analyzer (MAE-3000) also was used to determinate particle size of GeAs ultrafine particles.

The purity of condensate product can be analyzed by Inductively Coupled Plasma Emission Spectrometry (ICP-AES, IRIS Advantage 1000, THERMO, US). The final product obtained was dissolved completely in sealed digestion tube with aqua regia (HNO_3_:HCl = 1:3) at the 135 °C. The digested solution was dilute with water to 50 ml and used to determine the content of Ge by ICP-AES.

## Results and Discussion

### Factors on Evaporation Percent of GeAs ultrafine particles

In order to research the migratory rule of germanium in vacuum flash reduction and inert gas condensation process, four influencing factors were studied, including temperature, carrier gas flow rate, system pressure, and holding time.

#### Effects of temperature

**Figure 4:**
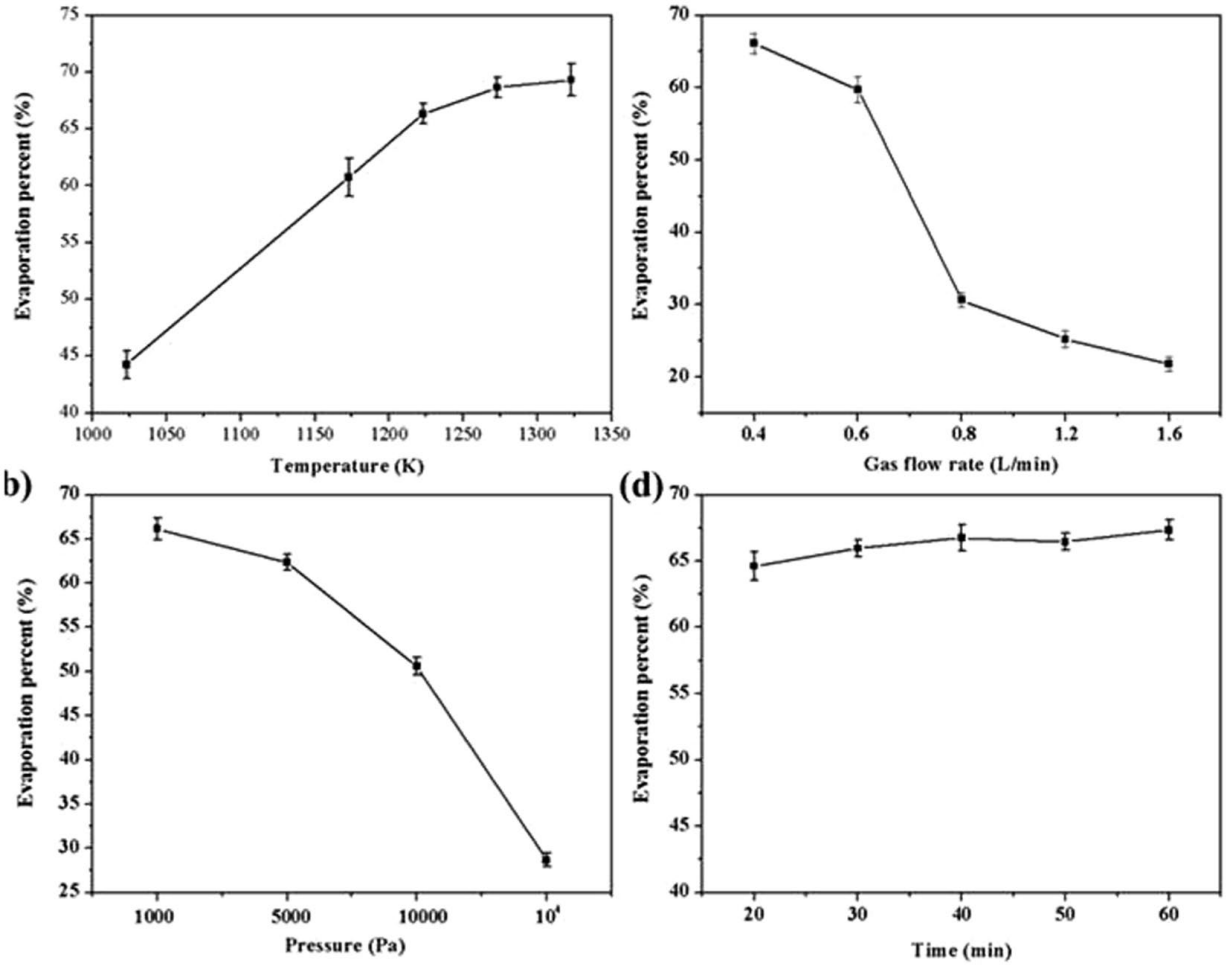
Effects of (**a**) temperature, (**b**) gas flow rate, (**c**) pressure and (**d**) holding time on evaporation percent of germanium.

A temperature range from 1023 to 1373 K, maintaining the reductant dosage at 10 wt. %, vacuum treatment time at 40 min, and nitrogen flow rate at 0.4 L/min corresponding to the system pressure of 1300 Pa, were chose to explore the germanium evaporation and formation of GeAs ultrafine particles. The relationship between evaporation percent and temperature was shown in Fig. 4(a). As can be seen from Fig. 4(a), evaporation percent of germanium in coal fly ash increased obviously with the increase of temperature. The evaporation percent of germanium increased from 44.26% to 69.32% when temperature increased from 1023 to 1373 K. A sharp increase appeared at 1223 K, where the evaporation percent of germanium jumped to 66.34% from 44.27% under the 1023 K. After temperature exceeding 1223 K, evaporation percent of germanium increased slowly. When temperature reached 1323 K, the evaporation percent can reach 69.32%. Obviously, temperature had significant effect on the evaporation of germanium. Hence, the temperature for maximal evaporation of germanium in coal fly ash was 1323 K.

#### Effects of carrier gas flow rate

The effect of gas flow rate on the evaporation percent was investigated in the range from 0.4 to 1.6 L/min when the temperature, system pressure, reaction time, reductant dosage was 1223 K, 1300 Pa, 40 min and 10 wt. %, respectively. The relationship between evaporation percent and carrier gas flow rate was shown in Fig. 4(b). The evaporation percent of germanium was 66.13% when the carrier gas flow rate was 0.4 L/min. However, evaporation percent of germanium presented downward trend extremely with an increasing carrier gas flow rate. For example, when the gas flow rate was 0.6 L/min, the evaporation percent of germanium was 59.73%. But the evaporation percent of germanium quickly dropped when the carrier gas flow rate increased to 0.8 L/min, which only was 30.63%. Its explanation is that increasing carrier gas flow rate can cause increase of partial pressure of evaporating substance, which would impede the evaporation of GeAs ultrafine particles. Therefore, the optimal carrier gas flow rate for evaporation of germanium in coal fly ash was 0.4 L/min.

#### Effects of system pressure

The experiments were conducted in the system pressure range from 1000 to 10^5^ Pa with temperature of 1223 K, carrier flow rate of 0.8 L/min, and holding time of 40 min. Figure 4(c) illustrated the effect of system pressure on evaporation percent. When system pressure was 1000 Pa, the evaporation percent of germanium was 66.07%. But the evaporation percent quickly dropped when the residue pressure increased to 1.0E + 05 Pa (atmosphere pressure), only 28.65% of germanium was recovered. Hence, when system pressure was 1000 Pa, the evaporation percent of germanium was optimal. We can also conclude that system pressure, which affects germanium evaporation velocity greatly, is another key factor for the germanium evaporation percent.

#### Effects of holding time

The effect of holding time was investigated from 20 to 60 min and experiments condition were conducted at 1223 K, 0.8 L/min and 1300 Pa with adding 10 wt. % carbon power. The relationship between evaporation percent of germanium and holding time is shown in Fig. 4(d). The result indicated that the effect of heating time for evaporation percent was not significant. When holding time was 40 min, the evaporation percent of germanium can reach to 66.75%. Therefore, the reaction time for recycling of germanium from coal fly ash can be controlled 40 min.

### Morphology Characteristic and Particle Size on GeAs Ultrafine Particles

In order to further understand formation of GeAs ultrafine particles in vacuum flash reduction and inert gas condensation, morphology, crystal structure and particle size distribution of these particles were studied under the condition of different temperature, carrier gas flow rate and system pressure.

#### Effects of temperature

**Figure 5:**
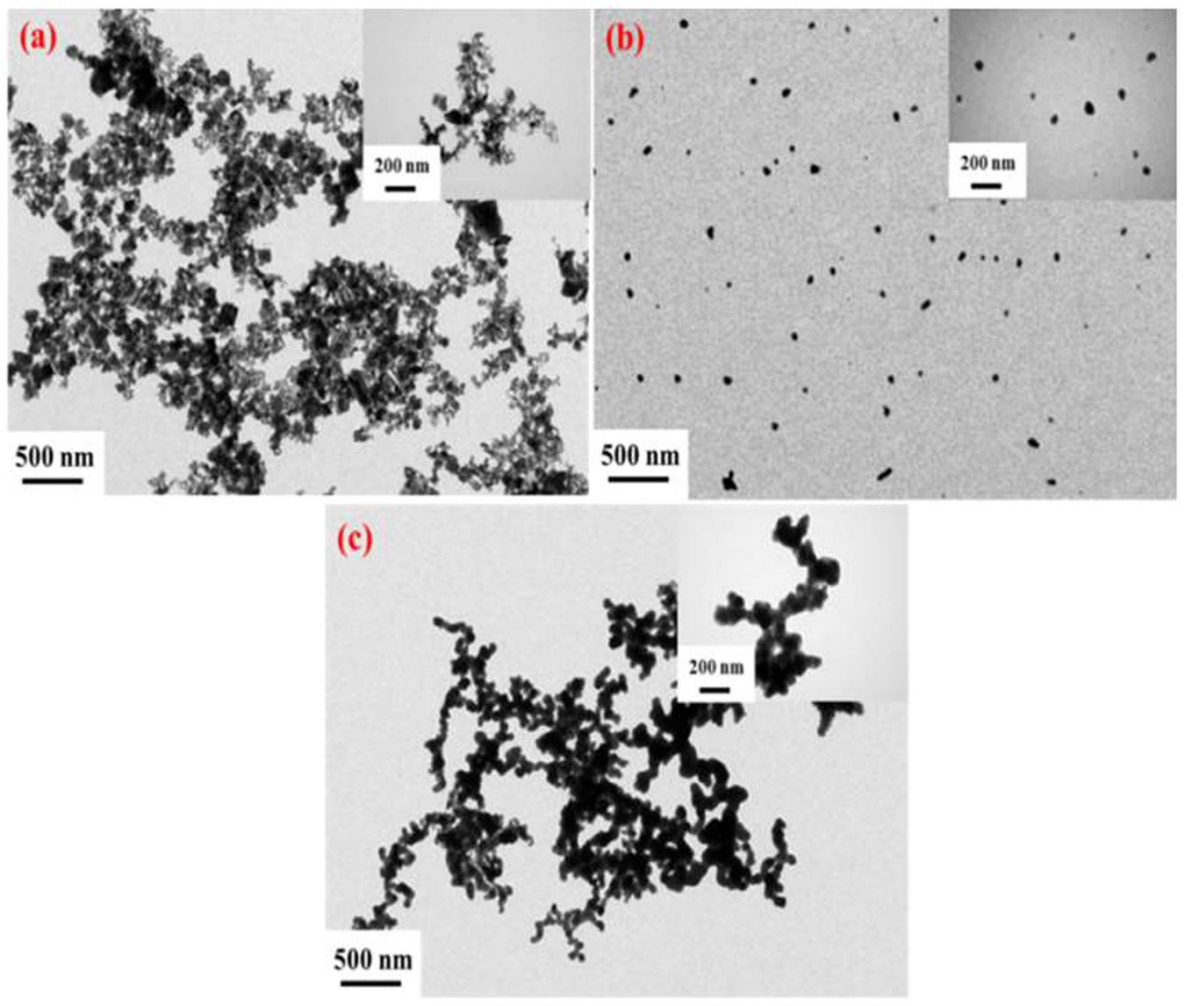
TEM pictures of GeAs ultrafine particles prepared at different temperatures (**a**) temperature = 1173 K; (**b**) temperature = 1223 K; (**c**) temperature = 1273 K.

Temperature played an important role on the size and morphology of the ultrafine particles. We observed the morphology of forming GeAs ultrafine particles at temperature of 1173 K, 1223 K and 1273 K by transmission electron microscopy (TEM). The morphologies of ultrafine particles have obvious differences when others condition was controlled at system pressure of 1300 Pa, holding time of 40 min, carrier gas flow rate of 0.8 L/min and 10 wt. % reductant dosage. TEM pictures showed that the morphology of forming ultrafine particles mainly presented cube particles under the temperature of 1173 K. Arrangement between particles were compact and aggregate (Fig. 5(a)). However, when temperature reached 1223 K, it appeared that most of the GeAs ultrafine particles prepared were dispersed and appeared spherical particles (Fig. 5(b)). The main particles size range is from 20 to 80 nm in diameter with an average diameter of 55.15 nm. Figure 5(c) showed that the TEM picture of GeAs ultrafine particles produced at 1273 K while other experimental conditions remained the same. Most of synthesized GeAs ultrafine particles were stuck together in cluster and formed agglomerate under the temperature 1273 K, which were generally irregular in shape. It can be explained by crystal growth of GeAs particles at high temperature resulting to these irregular shapes.

**Figure 6:**
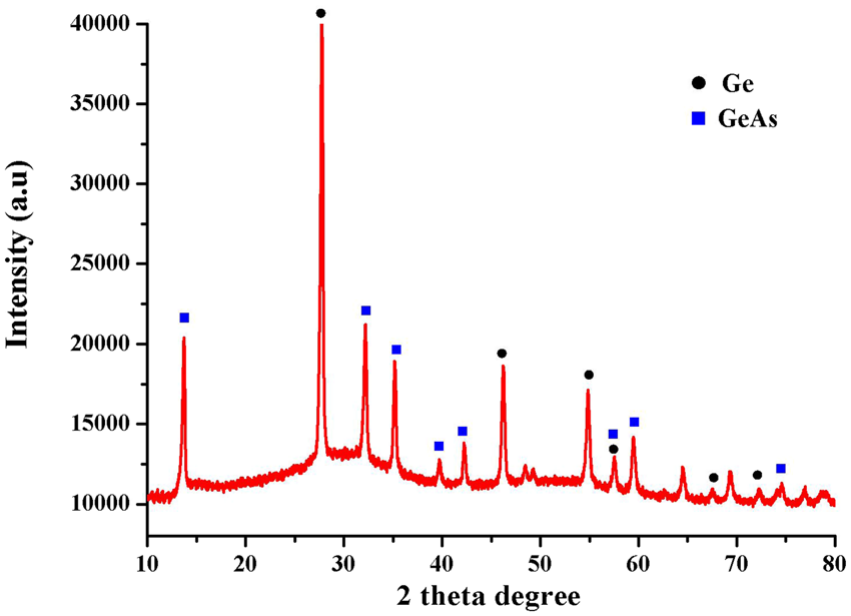
X-ray diffraction patterns of GeAs ultrafine particles.

Figure 6 showed the X-ray diffraction pattern of mineral phases of synthesized GeAs ultrafine particles with temperature of 1223 K. In synthesized GeAs ultrafine particles, the crystal peaks of them were sharp and the background signal was low indicating well-ordered crystalline materials. In forming GeAs ultrafine particles, crystal structure is mainly GeAs crystal shape. This suggested that GeAs crystal were well synthesized under temperature of 1223 K. Besides GeAs crystal, the crystal peak of metallic germanium also appeared in the ultrafine particles, which peak of metallic germanium phases at 27.5°, 46.8° and 55° (2θ) was sharp. However, with the increase of temperature, the crystal shape of synthesized GeAs crystal would be weakened gradually. However, when temperature increased 1273 K, the peak intensity of GeAs crystal phase was significantly decreased, as shown in SI Figure S1. This observation demonstrated that aggregation and growth of GeAs crystal caused the change of crystallinity with increase of temperature, which also consistent with the results of TEM observation.

#### Effects of carrier gas flow rate

**Figure 7:**
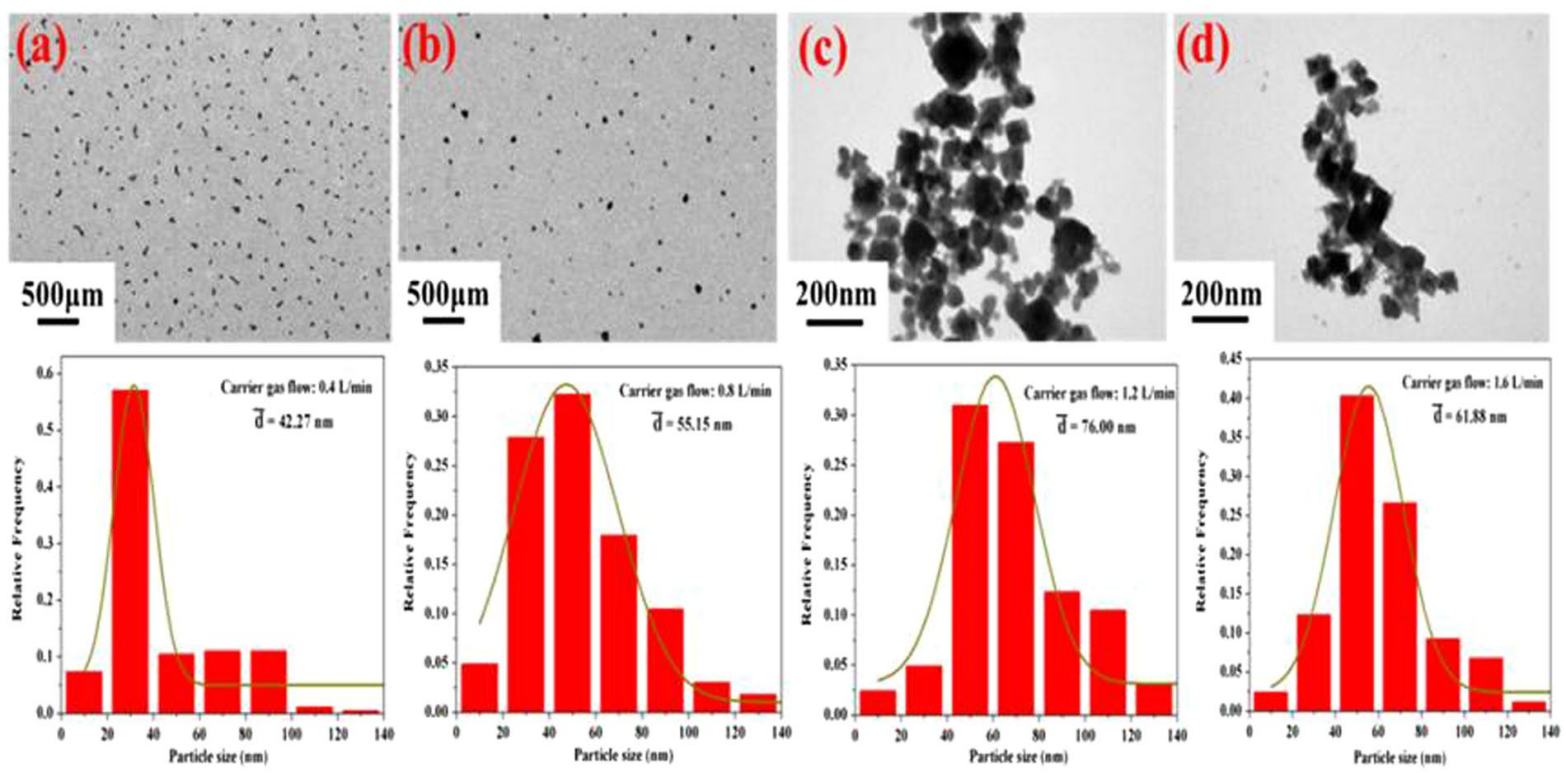
Morphologies and size distribution of the synthesized GeAs ultrafine particles with different nitrogen gas flow rate (**a**) 0.4 L/min; (**b**) 0.8 L/min; (**c**) 1.2 L/min; (**d**) 1.6 L/min.

Carrier gas flow rate was also key factor to affect the morphology and size distribution of GeAs ultrafine particles. Its morphology and size distribution was investigated using air flow rate in the range from 0.4 to 1.6 L/min, when others condition was controlled at temperature of 1223 K, system pressure of 1300 Pa, holding time of 40 min and 10 wt. % reductant dosage. Figure 7 showed the morphologies and size distribution of GeAs ultrafine particles at the carrier gas flow rate from 0.4 L/min and 1.6 L/min. Obviously, two completely different morphologies were presented for prepared ultrafine particles under different condition of carrier gas flow rate. Almost all ultrafine particles presented spherical microstructure when carrier gas flow rate was 0.4 and 0.8 L/min, but most of particles prepared at 1.2 and 1.6 L/min showed cubic morphology. All particles were evenly dispersed when the carrier gas flow rate was 0.4 L/min and 0.8 L/min, respectively. The particle sizes of nanoparticles prepared at carrier gas flow rate 0.4 L/min was mainly between 20 to 40 nm. The particles size below 40 nm in diameter accounted for 65.62% with the average diameter was 42.27 nm. The size distribution of nanoparticles prepared at carrier gas flow rate 0.8 L/min was more dispersed. The main particles size between 20 to 80 nm in diameter account for 77.5% with the average diameter was 55.15 nm. With the increase of carrier gas flow rate, the morphology of condensed ultrafine particles occurred change, but also particle sizes were increased compared with that of carrier gas flow rate 0.4 and 0.8 L/min. For the ultrafine particles prepared at the condition of carrier gas flow rate 1.2 and 1.6 L/min, their morphology presented mainly cube particles. In addition, their particle sizes were more dispersed and the average diameter was 76.00 and 61.88 nm, respectively. According to study of Wu *et al*.^46^, monodisperse GeO_2_ nanoparticles presented cube sharp and metallic germanium was spherical particles. This observation demonstrated that with increase of carrier gas flow rate, GeO_2_ in coal fly ash could not be involved in the reduction reaction and was directly blown away from the system. Hence, increasing carrier gas flow rate was disadvantage for synthesis of GeAs ultrafine particles.

#### Effects of system pressure

**Figure 8:**
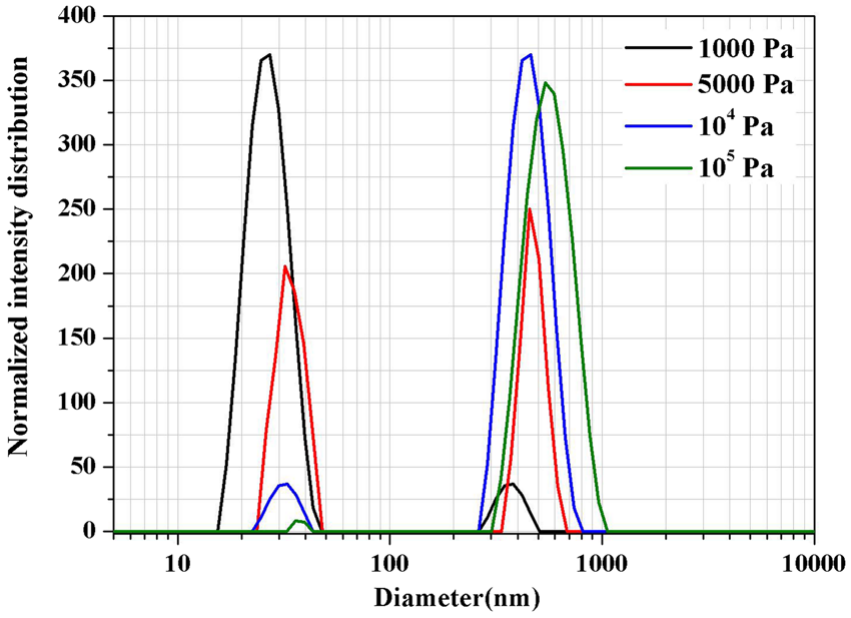
Size distributions of GeAs ultrafine particles under different pressure.

The influence of different pressure for synthesis of GeAs ultrafine particles was explored. The size distribution of the ultrafine particles under the condition of different pressure was measured by nanometer particle analyzer. The results were shown in Fig. 8. We can find that all ultrafine particles presented in two particle size ranges, namely, 10–100 nm and 100–1000 nm when others condition was controlled at temperature of 1223 K, nitrogen gas flow rate of 0.8 L/min, holding time of 40 min and 10 wt. % reductant, respectively. Interestingly, the number of particles in range of 10–100 nm presented a trend of gradual decrease with the increase of system pressure, according to the normalized intensity distribution curve of particles by determination of nanometer particle analyzer. For example, the size distribution of GeAs ultrafine particles was mainly range in 10–100 nm for condensate products under the condition of 1000 Pa. but when system pressure was increased to 10^5^ Pa, the size distribution of GeAs ultrafine particles was mainly range in 300–1000 nm. We can easy to draw the conclusion: low system pressure is advantage to form small particles of GeAs ultrafine particles. The reason is that lower system pressure can increase dispersion and dilution of vapor molecule.

**Figure 9:**
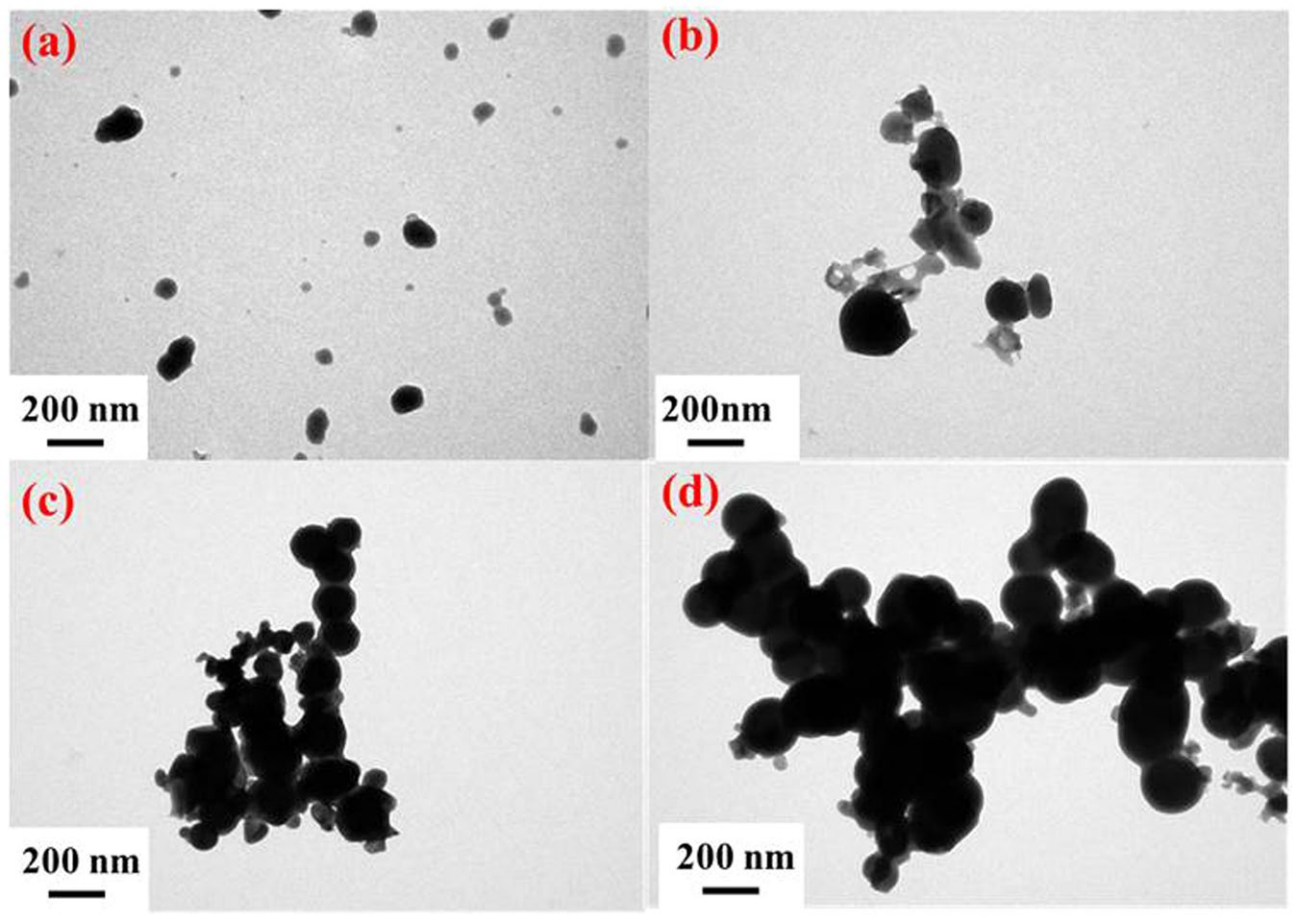
TEM pictures of GeAs ultrafine particles prepared at (**a**) system pressure = 1000 Pa; (**b**) system pressure = 5000 Pa; (**c**) system pressure = 10^4^ Pa; (**d**) system pressure = 10^5^ Pa.

TEM picture of GeAs ultrafine particles also verified the size distribution of germanium. Fig. 9(a–d) shows that the TEM picture of GeAs ultrafine particles obtained under system pressure of 1000 Pa and 10^5^ Pa when others condition was controlled at temperature of 1223 K, nitrogen gas flow rate of 0.8 L/min, holding time of 40 min and 10 wt. % reductant, respectively. When system pressure was 1000 Pa, most of the GeAs ultrafine particles were dispersed and presented spherical particles by TEM observation. With the increase of pressure, the GeAs ultrafine particles prepared began to aggregate and formed agglomerate of small particles. Their particle size began to increase gradually. It is the explanation that when system pressure was increased, abundant air in reaction system would hinder dispersion and condensation of GeAs ultrafine particles with carrier gas. Hence, it is hard to form dispersed particles.

Chemical composition of GeAs ultrafine particles were measured by ICP-AES. Germanium and arsenic were main elements in these particles, and besides germanium, few of Ca also were contained in them, which concentration was 2.60 wt. %. Although the prepared GeAs ultrafine particles still contained impurity Ca, further purification technologies for GeAs ultrafine particles to remove Ca.

By comprehensive result analysis of single factor experiments, morphology and the size distribution of GeAs ultrafine particles under different conditions, an optimal condition and evaporation percent of germanium is showed as follows: under the optimized parameters of 1223 K, 1000 Pa system pressure, 0.4 L/min carrier gas flow rate with 10 wt. % reductant for vacuum flash reduction and inert gas condensation process, the evaporation percent of germanium was 70.43 ± 0.86% (the value % ± error).

In addition, this technological process was compared with the traditional hydrometallurgy to recover germanium from the view of environment. For vacuum dynamic flash reduction and inert gas condensation process, the reductant dosage at 10 wt. % needs to be added in coal fly ash. Reaction conditions are strict and need high temperature and vacuum condition. For hydrometallurgical process, the liquid-solid rate (L/S) 5 acid can extract germanium in coal fly ash and reaction conditions and equipment are relatively simple (as shown in SI Table S1), but recovery ratio is medium. From the view of environment, waste acid and acid residues are significantly decreased, comparing with traditional recycling process. For example, adopting acid leaching method, 250 kg coal fly ash needed to use 1250 L hydrochloric acid, and germanium as form of GeO_2_ is extracted. After leaching reaction, large amount of waste acid and acid residues was identified as hazardous waste and needed treat and disposed in post-processing. But for this technology, no hazardous wastes were generated throughout the process. Hence, this technological process to recover germanium was feasible in aspect of environmental protection and technology.

### Synthesis Mechanism of Forming GeAs ultrafine particles

**Figure 10:**
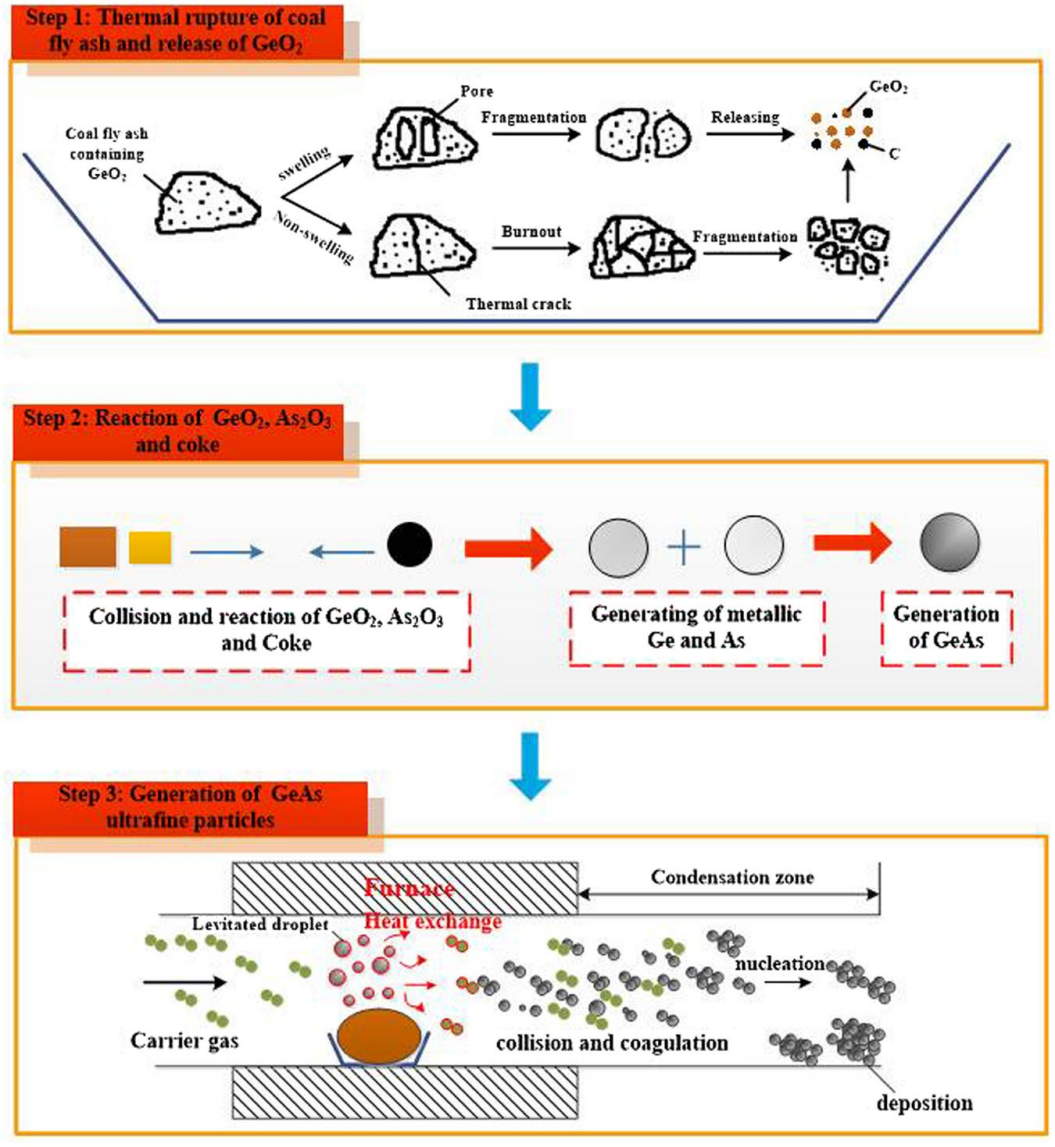
Mechanism of the formation of the GeAs ultrafine particles in the process of vacuum dynamic flash reduction and inert gas condensation.

A synthetic mechanism of GeAs ultrafine particles was described in Fig. 10. Firstly, the coal fly ash containing GeO_2_ expanded with heat cracking. After cracking of the coal fly ash, their matrix particles can either coalesce to form a large ash particle or produce several fine ash particles. And then the large and fine ash particles were burn out and formed char fragmentation at high temperatures^47, 48^. In this process, GeO_2_ particles would be released. Under the condition of high temperature and vacuum, a part of GeO_2_ can be evaporated from char fragmentation to enter gas phase, and the others still as form of gas phase entered the pore of char fragmentation from coal fly ash.

The second stage is reduction reaction of GeO_2_, As_2_O_3_ and coke. Reduction reaction of GeO_2_ and As_2_O_3_ entered to the pore of char fragmentation were occurred with coke by gas-solid phase reaction. Namely, evaporative GeO_2_ and As_2_O_3_ entered the pore of coke and triggered chemical reaction with a certain carrier gas flow. In this process, GeO_2_ was reduced metal germanium and meantime As_2_O_3_ was reduced arsenic. After the formation of metal Ge and As, a certain carrier gas flow rate in system is advantageous for evaporation of Ge and As. However, when the carrier gas flow rate exceeded a certain value, the residence time of GeO_2_ in reaction system was too short to hard to react with coke, and GeO_2_ was blown away quickly. In addition, increasing carrier gas flow rate is also disadvantageous for evaporation of Ge and As resulting from increasing partial pressure. When Ge and As entered into gas phase, conjugation reaction under the high temperature can be happen to generate GeAs. In addition, some other metal oxides also, such as Fe, Ca, and Al existing in coal fly ash could also be reacted with excess coke under vacuum condition. But their metals were hard to evaporate because the saturated vapor pressures of them were lower than that of Ge and As.

The synthesis mechanisms of ultrafine particles in the third stage based on inert gas consolidation can be described as three stages, including particle nucleation, growth, coagulation and coalescence^49–51^. Firstly, GeAs vapor and carrier gas heat transfer can occur in first stage. The energy of GeAs molecule gradually reduced because of the interaction between the ascending GeAs vapor and carrier gas. A supersaturated mixture is formed and meantime homogenous nucleation of GeAs vapor could occur. Once nucleate particles formed in the gas phase, they would coagulate at a rate that is proportional to the square of their number concentration. Temperature and carrier gas flow rate has significantly effect on the particle size of nanoparticles because GeAs particles can collide with carrier gas molecule and formed ultrafine particles cluster. Hence, this stage is particle growth. With the density of particles in gas phase increased resulting in higher the collision frequency between particles. Finally, under the condition of high supersaturation, coagulation and coalescence of particles could occur between different metallic clusters. With increasing the flow rate in a certain range, residence time of the condensed particles in the system decreases; therefore, the shorter time available for the particles results in a lower growth and consequently smaller particle size. In conclusion, temperature, carrier gas flow rate and vacuum pressure played a critical role in the formation and final size distribution of GeAs ultrafine particles.

## Conclusion

This study proposes a novel recycling process for germanium nanoparticles from coal fly ash. Thermodynamic calculation and saturated vapor pressure analysis showed that vacuum flash reduction process to recover germanium from coal fly ash is feasible. Inert-gas consolidation process can realize the preparation of germanium nanoparticles. The experimental results indicated that temperature, system pressure and carrier gas flow rate were the major factors on preparation of germanium nanoparticles. In addition, under the optimized parameters of 1323 K, 1000 Pa, 0.4 L/min carrier gas flow rate with 10 wt. % reductant, the evaporation percent of germanium was 70.43 ± 0.86% (the value % ± error). Finally, a three-step synthetic mechanism was clarified the process, namely, thermal rupture of coal fly ash, release of GeO_2_, reaction of GeO_2_ and coke, and preparation of enriched germanium nanoparticles. To sum up, this study developed a practical and feasible method to recycle germanium from coal fly ash with high added values.

## Electronic supplementary material

supporting information
