# Supplementary material for: One-Pot Synthesis of GeAs Ultrafine Particles from Coal Fly Ash by Vacuum Dynamic Flash Reduction and Inert Gas Condensation
Source: Sci Rep. 2017 Jun 16;7:3641. doi: 10.1038/s41598-017-03398-1 (PMC5473855; doi:10.1038/s41598-017-03398-1)
Supplement: Supplementary file 1 — supporting information [file 41598_2017_3398_MOESM1_ESM.pdf]

Supporting Information

One-Pot Synthesis of GeAs Ultrafine Particles from Coal Fly Ash by Vacuum Dynamic Flash Reduction and Inert Gas Condensation

Lingen Zhang and Zhenming Xu [*]

School of Environmental Science and Engineering, Shanghai Jiao Tong University, 800 Dongchuan Road, Shanghai 200240, People's Republic of China

Corresponding authors: Zhenming Xu, Tel: +86 21 54747495, Fax: +86 21 54747495, E-mail: zmxu@sjtu.edu.cn.

Supporting Information Content

3 Pages (including the cover page)

1 Figure

1 Table

Corresponding author: Zhenming Xu, Tel:+86 21 54747495; Fax:+86 21 54747495; E-mail：zmxu@sjtu.edu.cn

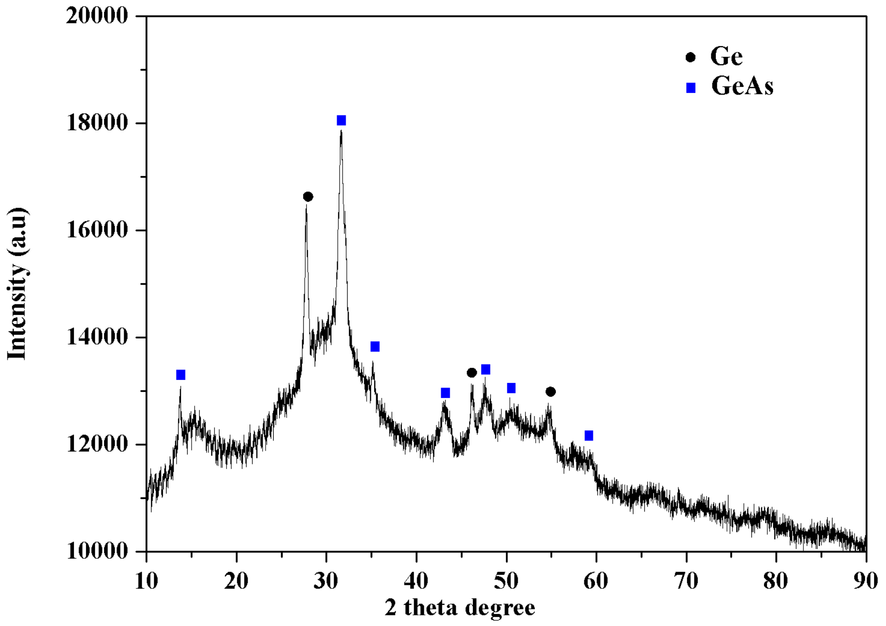

Figure S1 X-ray diffraction patterns of GeAs ultrafine particles under 1273 K

Table S1. Comparison of this technological process and traditional hydrometallurgical process

Technological process	Consumption of raw materials	Condition of reaction	Equipment	Secondary wastes
This technology	10 wt.% coke + coal fly ash	High temperature (950 ^{o}C)<br>High vacuum	Vacuum induction melting furnace	no hazardous wastes
Hydrometallurgical process	L/S 5 acid + coal fly ash	Relative low temperature (80-100°C)	Chemical reaction kettle	waste acids and acid residues
